# Candidate Reference Genes Selection and Application for RT-qPCR Analysis in Kenaf with Cytoplasmic Male Sterility Background

**DOI:** 10.3389/fpls.2017.01520

**Published:** 2017-09-01

**Authors:** Bujin Zhou, Peng Chen, Aziz Khan, Yanhong Zhao, Lihong Chen, Dongmei Liu, Xiaofang Liao, Xiangjun Kong, Ruiyang Zhou

**Affiliations:** ^1^Key Laboratory of Plant Genetics and Breeding, College of Agriculture, Guangxi University Nanning, China; ^2^Cash Crop Institute of Guangxi Academy of Agricultural Sciences Nanning, China; ^3^College of Biological and Food Science, Shangqiu Normal University Shangqiu, China

**Keywords:** cytoplasmic male sterility (CMS), reference genes, RT-qPCR, kenaf, gene expression, pollen development

## Abstract

Cytoplasmic male sterility (CMS) is a maternally inherited trait that results in the production of dysfunctional pollen. Based on reliable reference gene-normalized real-time quantitative PCR (RT-qPCR) data, examining gene expression profile can provide valuable information on the molecular mechanism of kenaf CMS. However, studies have not been conducted regarding selection of reference genes for normalizing RT-qPCR data in the CMS and maintainer lines of kenaf crop. Therefore, we studied 10 candidate reference genes (*ACT3, ELF1A, G6PD, PEPKR1, TUB, TUA, CYP, GAPDH, H3*, and *18S*) to assess their expression stability at three stages of pollen development in CMS line 722A and maintainer line 722B of kenaf. Five computational statistical approaches (GeNorm, NormFinder, ΔCt, BestKeeper, and RefFinder) were used to evaluate the expression stability levels of these genes. According to RefFinder and GeNorm, the combination of *TUB, CYP*, and *PEPKR1* was identified as an internal control for the accurate normalization across all sample set, which was further confirmed by validating the expression of *HcPDIL5-2a*. Furthermore, the combination of *TUB, CYP*, and *PEPKR1* was used to differentiate the expression pattern of five mitochondria F_1_F_0_-ATPase subunit genes (*atp1, atp4, atp6, atp8*, and *atp9*) by RT-qPCR during pollen development in CMS line 722A and maintainer line 722B. We found that *atp1, atp6*, and *atp9* exhibited significantly different expression patterns during pollen development in line 722A compared with line 722B. This is the first systematic study of reference genes selection for CMS and will provide useful information for future research on the gene expressions and molecular mechanisms underlying CMS in kenaf.

## Introduction

Cytoplasmic male sterility (CMS) is a maternally inherited trait and is widely used in hybrid seed production in many crops (Schnable and Wise, 1998; Li et al., 2014). The phenotype of a CMS plant is similar to that of normal plant but differs in the lack of functional pollen. In flowering plants, pollen development is a complex process that contains a wide variety of genes (Sun et al., 2013). These genes are mainly involved in energy metabolism, stamen development (B-class and C-class), and biosynthesis. Abnormal expression of these genes causes loss of pollen function. For example, the expression of *DcMADS2* and *DcMADS3*, which are homologs genes with B-class, were significantly down-regulated in “carpeloid” CMS flower of carrot. Research findings show that specific cytoplasmic (mitochondrial) influence the expression of MADS box genes for B-activity during early differentiation of petals and stamens leading to the “carpeloid” CMS phenotype (Linke et al., 2003). Thus, the analysis of the expression patterns of key genes that are involved in pollen development is useful to explore the molecular regulatory mechanisms underlying plant CMS and pollen development.

Numerous methods have been used to investigate gene expression analysis, such as: northern blotting, semi-quantitative reverse transcription PCR, in situ hybridization, real-time quantitative PCR (RT-qPCR), and RNase protection. The RT-qPCR technology is used worldwide for gene expression analysis due to its high sensitivity, specificity, accuracy, and broad quantification range (Bustin et al., 2005; Coito et al., 2012). However, multiple non-specific variations affect the reliability of gene expression analyses, such as RNA quality, amount of input RNA, primer specificities and the efficiency of RNA reverse transcription (Udvardi et al., 2008). RT-qPCR data must be normalized using a reference gene to avoid these non-specific variations (Radonic et al., 2004; Huggett et al., 2005).

The ideal reference gene must have transcription abundance similar to that of the target gene under variable conditions and must not be co-regulated with the target gene (Radonic et al., 2004). Housekeeping genes are considered to be stably expressed regardless of the organism's developmental stage or environmental conditions, and they have been considered as ideal reference genes. Currently, a number of traditional housekeeping genes have been widely used as reference genes for the normalization of quantitative gene expression, including *18S* (Xue et al., 2012), *ELF1A* (Marum et al., 2012; Yue et al., 2016), *GAPDH* (Zhao et al., 2013), and *CYP* (Qi et al., 2010). Nevertheless, studies have shown that no single reference gene can always maintain stability in response to changes in the environmental conditions, as noted with *18S* (Wan et al., 2010), *ELF1A*(Xu et al., 2014), *GAPDH* (Tong et al., 2009; Wang C. et al., 2016) and *CYP* (Wei et al., 2013; Yeemin et al., 2016). Consequently, a systematic selection and validation of candidate reference genes in each experimental condition should be performed before selecting a reference gene for the normalization of data in a gene expression analysis.

Kenaf (*Hibiscus cannabinus* L.) is an annual fiber crop having versatile applications e.g., used in cordage, sacking, paper, building materials, animal bedding, carpet backing (Zhao et al., 2013). Kenaf spontaneous CMS mutant “UG93” and several CMS lines have been reported (Zhou et al., 2008). The CMS lines have been utilized in hybrid seed production for kenaf crops, but little is known regarding the molecular mechanism of the CMS. To understand the molecular mechanisms underlying of kenaf CMS, our previous work had been performed to compare the transcriptional differences between the CMS and its maintainer lines using RNA-seq (Chen et al., 2014), and many differentially expressed genes were cloned including *cox3, HcPDIL5-2a, HcMADS-box*, and *MYB21* (Chen and Zhou, 2011; Jin et al., 2011; Chen et al., 2012; Qian et al., 2016). Analyzing the expression patterns of genes of interest might aid our understanding of the molecular mechanisms underlying CMS in kenaf crop. However, there is no systematic study conducted for reliable reference gene selection at different pollen development phases in kenaf, which limits further the differential gene studies and transcription analysis.

Therefore, this study assessed the expression stabilities of 10 candidate reference genes (*ACT3, ELF1A, G6PD, PEPKR1, TUB, TUA, CYP, GAPDH, H3*, and *18S*) for the normalization of data at three pollen developmental stages in CMS line 722A and maintainer line 722B, using five statistical algorithms. The expression stability of potential reference genes was validated by normalizing the RT-qPCR data with *HcPDIL5-2a*. Furthermore, the expression patterns of five mitochondria F_1_F_0_-ATPase subunit genes *atp1, atp4, atp6, atp8*, and *atp9*, in lines 722A and 722B of kenaf were examined using the newly identified stable reference genes.

## Materials and methods

### Plant materials

**Figure 1:**
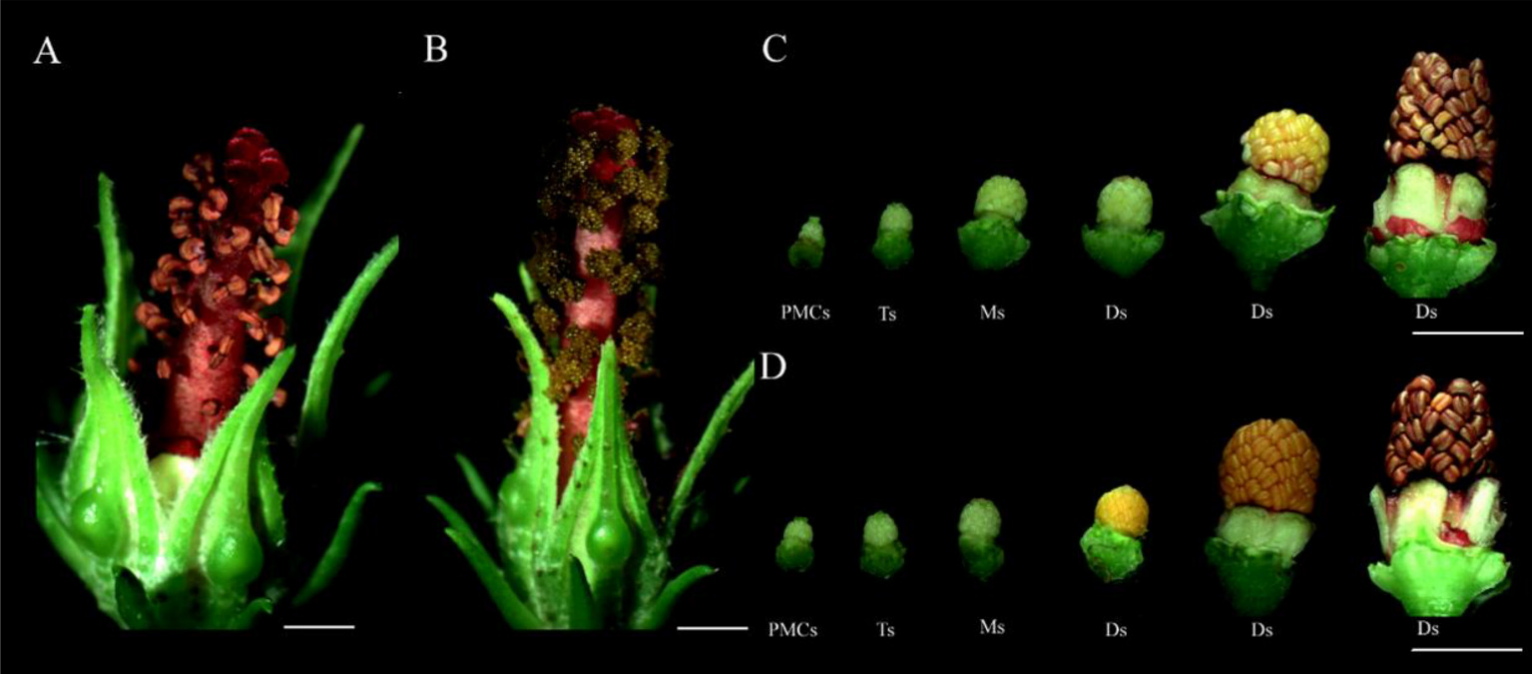
Phenotypic characterization of the CMS line 722A and its maintainer line 722B's flower buds. **(A,C)** Phenotypes of the CMS line's floral buds; **(B,D)** phenotypes of the maintainer line's floral buds. PMCs, The flower buds of pollen mother cell stage; Ts, The flower buds of tetrad stage; Ms, The flower buds monokaryotic pollen stage; Ds, The flower buds of dual-core pollen stage. Bar = 0.5 cm.

In this study, kenaf CMS line 722A and its maintainer line 722B were used. Both lines 722A and 722B had similar nuclear genetic background, but with different cytoplasm. Lines 722A and 722B were sown in the experimental field of Guangxi University under natural conditions in the year 2015. Standard cultural practices (irrigation, weeding, insecticide, and fertilization) were carried out according to the crop's demand over the whole growth period. The line 722A plants had floral organs with aberrant anthers (Figures 1A, C). However, line 722B plants had floral organs with normal anthers, and mature pollen grains were produced during pollen development phasse (Figures 1B, D). Anther samples were collected at tetrad (floral bud with length of 2.5–3.5 mm), monokaryotic (3.5–6.0 mm) and dual-core (greater than 6.0 mm) stages of pollen development in lines 722A and 722B, which represented the early, middle and late stages of pollen abortion in line 722A, respectively. Plant samples were quickly frozen in the liquid nitrogen and stored at −80°C for RNA extraction. Three replications were used and each replicate comprised of 30 individuals.

### Semi-thin sections and microscopy

Different lengths of floral buds from the CMS and its maintainer lines were placed in a cold Carnoy's fixative solution (ethanol: acetic acid = 3:1) for 24 h at 4°C. The floral buds were dehydrated using graded ethanol series (Lee et al., 2008). Dehydrated floral buds were embedded in paraffin and sectioned into 12-μm thick slides. Serial sections of the floral bud tissues were mounted on slide glass and stained with pissophane-hematoxylin. Sectioned floral buds were observed and photographed through microscope (Leica DMI3000B).

### RNA extraction and cDNA synthesis

Total RNA was extracted from each of the samples using the Quick Plant RNA Isolation Kit (Huayueyang, Beijing, China, Cat. No: 0416-50GK). DNase I (RNase free) (TransGen, Beijing, China, Cat. No: GD201-01) was used at 37°C for 30 min to isolate gDNA. The RNA integrity was examined by 1% agarose gel. The concentration and quality of the total RNA were measured using Nanodrop™ 2000 spectrophotometer (Thermo Fisher Scientific, Waltham, MA, USA). cDNA was synthesized using 2,000 ng of high-quality total RNA and the PrimeScript™ RT reagent Kit (TaKaRa, Dalian, China, Cat. No: RR037A). The cDNA was synthesized according to the kit's protocol and subsequently diluted 5-fold with molecular biology grade sterile water.

### Primer design

Total, 10 commonly used reference genes, *ACT3, ELF1A, G6PD, PEPKR1, TUB, TUA, CYP, GAPDH, H3*, and *18S*, were identified from the published literature. The corresponding gene sequences were cloned and sequenced based on kenaf transcriptome sequencing data (Chen et al., 2014), and the corresponding gene sequences has been submitted to NCBI (www.ncbi.nlm.nih.gov/). On the basis of the corresponding gene sequences, primers were designed for RT-qPCR using the Primer 3 Plus program (www.primer3plus.com/cgi-bin/dev/primer3plus.cgi). The primers of corresponding gene were tested using Primer Select program within the Lasergene 9 software (Burland, 2000) for dimerization and hairpin formation.

### Standard curve construction and RT-qPCR

Standard curves were generated using a 5-fold dilution series of the linearized TA-cloning plasmid containing sequences of candidate reference genes and target genes. The correlation coefficient (R^2^) and slope for each gene were estimated using standard curves. The slope was used to assess the amplification efficiency using the formula E = 10 ^(−1/slope)^ −1 (Pfaffl et al., 2004). RT-qPCR reactions were performed in a CFX96 Real Time PCR System (Bio-Rad, California, USA) using a SYBR Premix Ex Taq™ Kit (TaKaRa, Cat. No: RR820A). Each RT-qPCR system contained 1.5 μl of template, 40 nM each primer, and 7.5 μl of SYBR Green PCR master mix, having volume of 15 μl. The RT-qPCR conditions were as follows: 30 s at 95°C and 40 cycles of 15 s at 95°C for denaturation and 30 s at 60°C for annealing. Fluorescence was measured at 65–95°C. Agarose gel electrophoresis and sequence were performed for the amplified products' specificity analysis. All RT-qPCR reactions were performed in triplicate technical replicates.

### Candidate reference gene stability analysis

Four common statistical packages, GeNorm (Vandesompele et al., 2002), NormFinder (Andersen et al., 2004), ΔCt (Silver et al., 2006), and BestKeeper (Pfaffl et al., 2004), were implemented to estimate the stability of each candidate reference gene.

For GeNorm and NormFinder, the *Ct*-values were transformed into relative quantities using the formula 2^−ΔCt^; ΔCt is the corresponding *Ct*-value minus the minimum *Ct*-value (Chen et al., 2015). GeNorm was used to analyze gene stability (*M*-value) and pairwise variation (V_n_/_n+1_) value by pairwise comparisons among all of the reference genes. The *M*-value correlated with the expression stability, and the lowest *M*-values were considered the most stable. The threshold of 0.15 was the criterion value of V_n_/_n+1_. A value less than 0.15 are not necessary to use a reference gene for the normalization of the RT-qPCR analysis (Vandesompele et al., 2002). The NormFinder approach is used to rank the candidate reference genes by estimating intra- and inter-group gene expression variations (Andersen et al., 2004). The reference gene with the lowest value was also considered to be the most stable. The ΔCt approach, which compares standard deviation (SD) values of each gene, was used to rank the candidate reference genes (Silver et al., 2006). BestKeeper ranked the candidate reference genes using the SD and the coefficients of variance (CVs), and the lowest SD value indicated the most stable gene. Based on the ranking from the four statistical approaches, RefFinder (http://www.leonxie.com/referencegene.php) assigned an appropriate weight to each gene and calculated the geometric mean of their weights for the overall final ranking (Kim et al., 2003).

### Validation of reference genes

The target gene protein disulfide isomerase-like 5-2a (*HcPDIL5-2a*), was up-regulated in the CMS line compared with maintainer line of kenaf (Jin, 2011), was used to validate the reliability of the potential reference genes determined by RefFinder and GeNorm approaches across all sample sets. The relative expression levels of *HcPDIL5-2a* at three stages of pollen development in lines 722A and 722B were normalized with four different reference gene strategies: (1) the most stable reference gene; (2) the geometric mean of the two most stable reference genes; (3) the geometric mean of the three most stable reference genes; and (4) the least stable reference gene. The relative expression of the target gene was calculated according to 2^−ΔΔCt^ method. Student's *t*-test (*P* ≤ 0.05) was applied to the variance analysis.

### Expression pattern analysis of ATP synthase subunit genes

The combination of *TUB, CYP*, and *PEPKR1*, which was recommend by the RefFinder and GeNorm methods across all sample sets, was used as an internal control to differentiate the expressions of five mitochondria F_1_F_0_-ATPase subunit genes (*atp1, atp4, atp6, atp8*, and *atp9*) in lines 722A and 722B. The expression patterns of these genes were calculated using 2^−ΔΔCt^ method. Least significant differences were determined by Duncan's test (*P* ≤ 0.05) using SPSS Statistics 21.0 (IBM).

## Results

### Determining the stage of anther collection based on a cytological analysis

**Figure 2:**
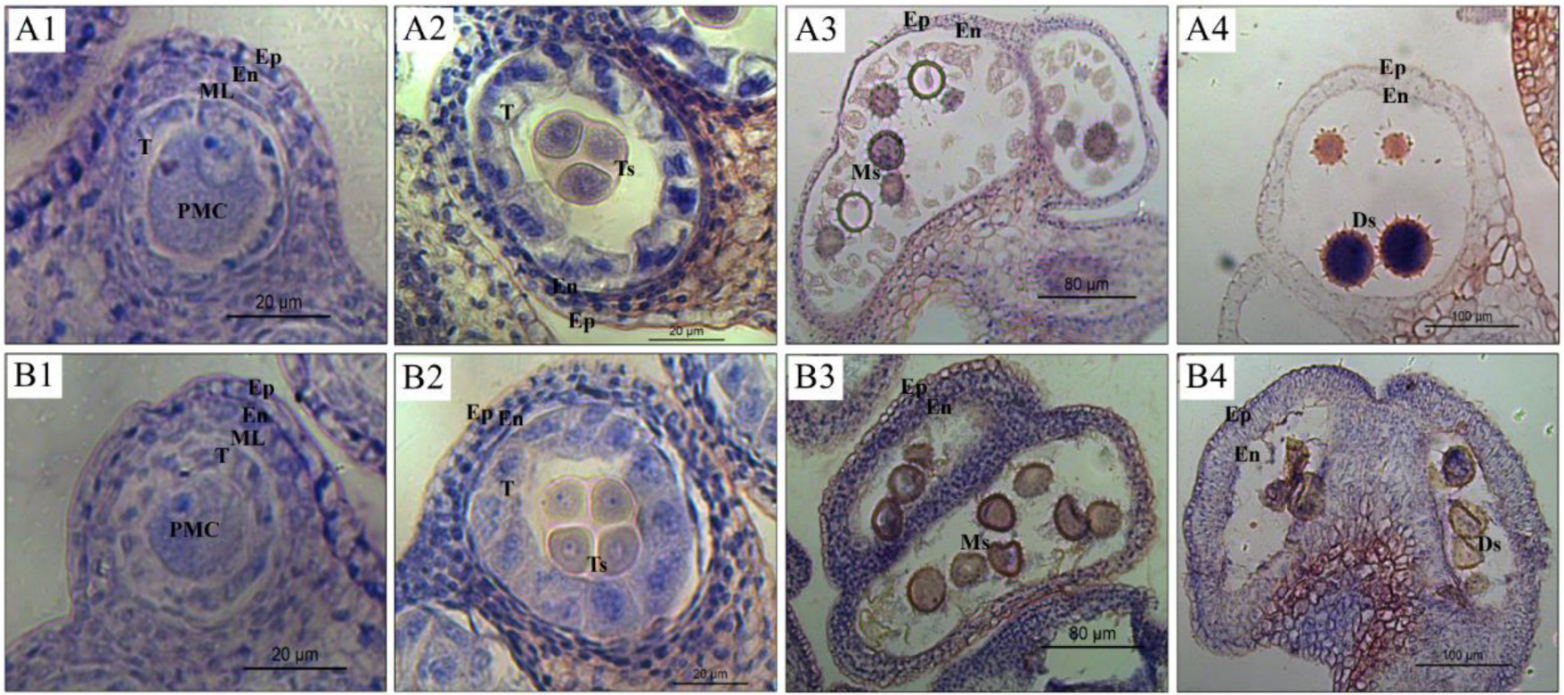
Micrographs of anthers at different developmental stages in the maintainer line 722B **(A1–A4)** and CMS line 722A **(B1–B4)**. **(A1–B4)** Indicate anther of pollen mother cell, tetrad, monokaryotic, and dual-core stage respectively. The Ep, En, ML, T, PMC, Ts, and Ms, Ds represent epidermis, endothecium, middle layer, tapetum, pollen mother cell, microspore of tetrad, monokaryotic and dual-core stage respectively.

In order to determine the anther collection stage, pollen development at different stages were observed in CMS line 722A and maintainer line 722B of kenaf (Figure 2). The results revealed that the pollen abnormality was initiated after the tetrad stage in line 722A, although no obvious difference was observed between lines 722A and 722B from the pollen mother cell (PMC) to tetrad stage. At the PMC stage, the PMCs were surrounded by the tapetum, and the anther parietal cells were differentiated into the epidermis, endothecium, middle layer and tapetum (Figures 2A1, B1). At the tetrad stage, PMCs formed tetrads after two rounds of meiosis and were wrapped by callose, and tapetum cells expanded and then condensed (Figures 2A2, B2). However, most of the pollen was seriously distorted in line 722A and only a small portion of the pollen was structurally intact compared with line 722B at the monokaryotic stage (Figures 2A3, B3). At the dual-core stage, the microspores were malformed in line 722A (Figure 2A4), although pollen development was normal, the cytoplasm was condensed, and fertile pollen spores were produced in line 722B (Figure 2B4). Based on the cytological analysis of pollen development in lines 722A and 722B, the anther samples were collected at tetrad, monokaryotic and dual-core stages in lines 722A and 722B,that represented the early, middle and late stages of pollen abortion, respectively, in line 722A.

### Evaluation of PCR primer specificity and amplification efficiency

The specificity of the primer was identified by the presence of a single DNA fragments with the expected size for each primer pair in 3% agarose gel electrophoresis (Figure S1). These DNA fragments were cloned and sequenced, and the sequences had high identities with expected sequences (Supplementary Material). Additionally, the primer specificity was further validated by the presence of a single peak in the melting curve analysis (Figure S2). Subsequently the amplification efficiencies (E) and *R*^2^ values were calculated with the standard curve of each primers pair. The *E*-values of these genes ranged between 0.902 and 1.003, and the *R*^2^ between 0.990 and 1.000 (Table 1). These results demonstrated that the primers of these genes were reliable.

**Table 1:** The amplicon characteristics of primers, 10 candidate reference genes and 6 target genes.

**Gene name (abbreviation)**	**Accession NO.**	**Forward primer sequence (5′-3′)/Reverse primer sequence (5′-3′)**	**PCR Efficiency**	***R*^2^**	**Product size (bp)**
18S ribosomal RNA (*18S*)	KX816334	AGAAACGGCTACCACATC/TACTCATTCCAATTACCAGACTC	0.953	0.999	122
Actin 3 (*ACT3*)	KX689256	GTGAGGATATTCAACCCCTTGTCT/CATCTTTCTGTCCCATACCAACC	0.983	0.999	150
Cyclophilin (*CYP*)	KX689255	TCATCTGCACCGCCAAAA/CTTTCTCCACGGCTCTCACC	0.928	0.999	96
Eukaryotic elongation factor 1-alpha (*ELF1A*)	KX703003	GAACATGATCACGGGGACCT/GAGTGAAGGCAAGCAGAGCA	0.932	0.999	128
Glucose-6-phosphate dehydrogenase (*G6PD*)	KX703004	ACGAATTCTCGAAAAGTAGCCAAG/TCCGAGTCCATCCACCAAG	0.972	0.999	141
Glyceraldehyde-3-phosphate dehydrogenase (*GAPDH*)	KX783037	AACGAAAAGGAATACAAGCCAGAG/AAGACCCTCAACAATGCCAAA	0.924	0.999	117
Histone 3 (*H3*)	KX703005	GTGGAGTCAAGAAGCCTCACAG/ATGGCTCTGGAAACGCAAA	0.902	0.999	164
Phosphoenolpyruvate Carboxylase-Related Kinase 1 (*PEPKR1*)	KX703006	TGCCATGAGAATCGCCAAC/GGACACCAACCAAAAGCACA	1.003	0.999	149
Alpha-tubulin (*TUA*)	KX703007	ATTGGCGGAGGTGATGATG/TGGAAGAGTTGGCGGTATGTT	0.936	0.999	140
Beta-tubulin (*TUB*)	KX703008	TTTTCCGACCCGACAACTTC/AGTTCCGCTCCTTCCGTGT	0.930	0.999	85
Protein disulfide isomerase-like5-2a of kenaf (*HcPDIL5-2a*)	HQ638208	CGTTGCTCCTGATGTGTCTATTCT/CTGTCTCATTCAAGCCAAAACCT	0.913	0.999	110
ATP synthase subunit 1 (*atp1*)	KC686836	TAAAAGCGGTAGATAGCCTGG/AATCGCTACATAGACACAATACAA	0.909	0.999	173
ATP synthase subunit 4 (*atp4*)	MF511050	TCTTCCCATCGCATCCGTCTT/TTATTCTTACCCCACCCGAACCAT	0.926	1.000	159
*ATP synthase subunit 6 (atp6)*	HM535783	GCTAATCTCTTATTGTTTTCGCGCA/ATAGCATAGTCCAAGCGAAGCCAC	0.934	1.000	113
ATP synthase subunit 8 (*atp8*)	KM281806	TTCTGGTTATGCCTTTTCTCTT/TGTTTCTTTCTATTCCGCGTGAG	0.942	1.000	319
ATP synthase subunit 9 (*atp9*)^a^	JX206830	ATGAATGATAAAGCGCGTGACGAG/CGGTTAGAGCAAAGCCCAAAATG	0.995	1.000	208

a *The primers sequences of this gene was according to Zhao et al. (2013)*.

### Expression analysis of the candidate reference gene

To assess the influence of pollen development on the expression stability of the candidate reference genes, the RT-qPCR data of 18 samples were divided into three sets. The first set included all of the samples. The second set contained nine samples from 722A, and the third set was composed of nine samples from 722B.

**Figure 3:**
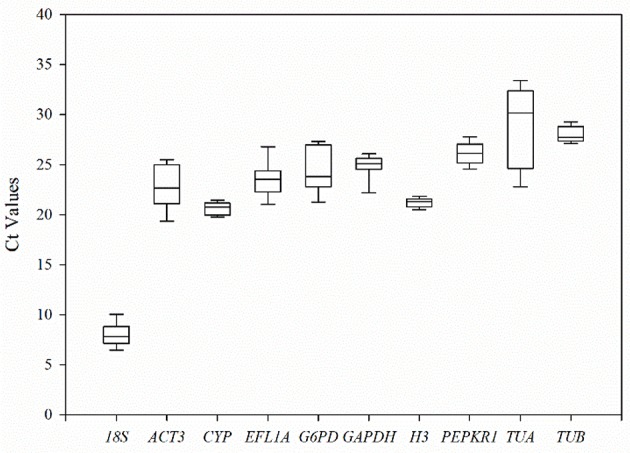
The threshold cycle (Ct) distribution of each candidate reference genes in all experimental samples. A lines across the box of Ct value represent the median values. Lower and upper boxes show the 25 and 75% percentiles. *Whiskers* represent the maximum and minimum Ct values. The y-axis represents the Ct values of three biotechnology replicates of all experimental samples, while the x-axis represents the 10 candidate reference genes.

The transcription levels of 10 candidate reference genes were detected by RT-qPCR assays at three stages of pollen development (tetrad, monokaryotic and dual-core) in lines 722A and 722B of kenaf (Figure 3; Table S1). The *Ct*-values indicated that the transcripts of these genes were present in a relative widely abundance range, from 6.06 to 33.79, in the 18 tested samples. Among them, *TUA* showed the lowest transcription abundance, with a mean *Ct*-value of 28.89, whereas *18S* showed the highest abundance, with a mean *Ct*-value of 7.97. The transcription levels with the least variation were displayed by *TUB, CYP, PEPKR1*, and *H3* (*SD* < 1.20), while *TUA* was the most variable (*SD* = 3.92).

### Stability ranking of candidate reference genes

To evaluate the expression stability of the candidate reference genes at the three stages of pollen development in lines 722A and 722B, four statistical approaches, GeNorm, NormFinder, ΔCt and BestKeeper, were used.

**Figure 4:**
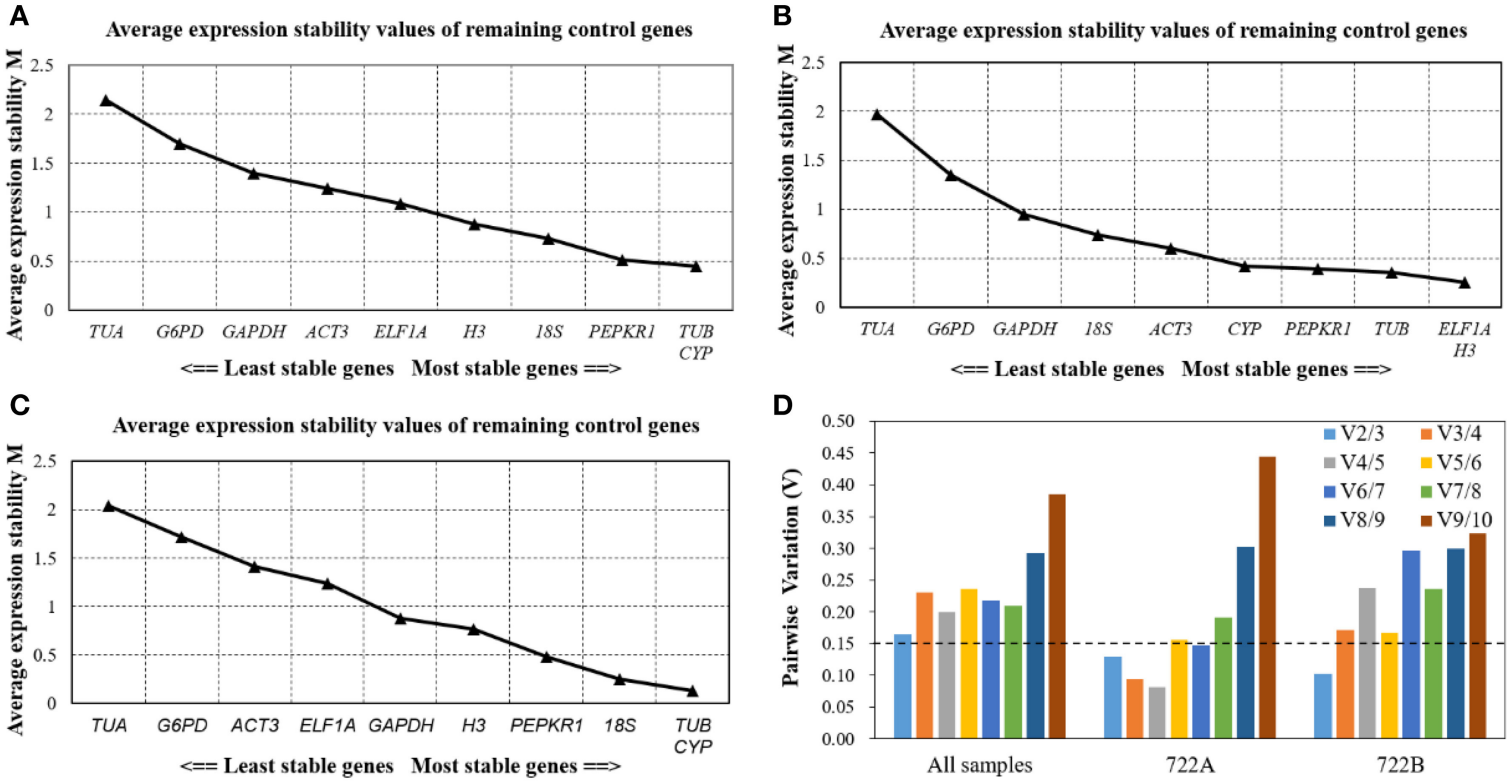
Stability ranking and pairwise variation (V) analysis of the 10 candidate reference genes in different sample sets using GeNorm. The average expression stability values (M) of the 10 candidate reference genes performed in all sample **(A)**, 722A sample set **(B)**, 722B sample set **(C)**, The most stable genes are on the right, and the least stable gene are on the left. The optimal number of reference genes required for accurate normalization **(D)**. GeNorm was used to analyze pairwise variation (V_n_/V_n+1_) between the normalization factors NF_n_ and NF_n+1_, *V*-value < 0.15 denotes that additional reference genes will not significantly improve the accuracy of the results.

GeNorm determines stability rankings based on *M*-values of candidate reference genes. The lowest *M*-value indicates the most stable expression, while the highest *M*-value indicates the least stable expression. The most stable candidate reference gene was not the same in the three sample sets, but the least stable candidate reference gene was consistently *TUA* with *M*-values of 2.15, 1.97, and 2.04 (Figures 4A,C). In all of the sample sets and the 722B sample set, *TUB* and *CYP* were the most stable candidate reference genes, with *M*-values of 0.44 and 0.13, respectively (Figures 4A,C). In the 722A sample set, *ELF1A* and *H3* were the most stable reference genes, with *M*-values of 0.26 (Figure 4B).

Additionally, GeNorm was used to calculate the optimal number of candidate reference genes using the pairwise variation (V_n_/_n+1_) between two sequential normalization factors (NFn and NFn+1). In this study, when all of the samples were combined, the pairwise variation values were above the proposed 0.15 cut-off, and V_2/3_ was the lowest with *V*-values of 0.165 (Figure 4D). This result suggested that at least three candidate reference genes were required for the normalization of a gene expression analysis. However, if the value of V_2/3_ was below 0.15 in the 722A and 722B sample sets, then two reference genes were sufficient for normalizing RT-qPCR data (Figure 4D).

The NormFinder approach calculates gene expression stability based on their minimal combined inter- and intra-group expression variations, which are based on normalization factor calculations. The expression stability of 10 candidate reference genes was calculated with NormFinder (Table 2). In all of the samples and the 722B sample sets, *TUB* was the most stable candidate reference genes. In the 722A sample set, *ELF1A* was the most stable candidate reference genes.

**Table 2:** Stability ranking of 10 candidate reference gene using NormFinder.

**Rank**	**All samples**		**722A**		**722B**	
	**Gene**	**Stability**	**Gene**	**Stability**	**Gene**	**Stability**
1	*TUB*	0.110	*ELF1A*	0.067	*TUB*	0.141
2	*CYP*	0.113	*H3*	0.115	*CYP*	0.172
3	*PEPKR1*	0.136	*TUB*	0.120	*18S*	0.215
4	*ELF1A*	0.192	*PEPKR1*	0.174	*ELF1A*	0.249
5	*18S*	0.184	*CYP*	0.205	*GAPDH*	0.253
6	*H3*	0.195	*ACT3*	0.236	*PEPKR1*	0.252
7	*ACT3*	0.198	*18S*	0.254	*ACT3*	0.271
8	*G6PD*	0.222	*GAPDH*	0.316	*H3*	0.301
9	*TUA*	0.223	*TUA*	0.318	*TUA*	0.338
10	*GAPDH*	0.247	*G6PD*	0.363	*G6PD*	0.468

The ΔCt approach identifies gene expression stability based on the mean *SD* values of each gene set of pairwise combinations. For all of the samples and the 722B sample set, *TUB* was the most stable candidate reference gene (Table 3). However, for 722A sample set, *H3* was the most stable candidate reference gene (Table 3).

**Table 3:** Stability ranking of 10 candidate reference gene using ΔCt.

**Rank**	**All samples**		**722A**		**722B**	
	**Gene**	**Mean SD**	**Gene**	**Mean SD**	**Gene**	**Mean SD**
1	*TUB*	1.486	*H3*	1.301	*TUB*	1.420
2	*CYP*	1.542	*TUB*	1.319	*CYP*	1.440
3	*PEPKR1*	1.555	*ELF1A*	1.334	*18S*	1.497
4	*H3*	1.796	*PEPKR1*	1.341	*PEPKR1*	1.601
5	*ELF1A*	1.916	*CYP*	1.423	*GAPDH*	1.828
6	*ACT3*	2.158	*18S*	1.595	*H3*	1.907
7	*GAPDH*	2.159	*ACT3*	1.735	*ELF1A*	2.112
8	*G6PD*	3.129	*GAPDH*	1.988	*ACT3*	2.182
9	*18S*	3.504	*G6PD*	3.201	*G6PD*	3.091
10	*TUA*	3.946	*TUA*	4.460	*TUA*	3.328

BestKeeper relies on a pairwise correlation analysis of each gene, the lowest *SD* values indicated the most stable gene. The highest ranking candidate reference gene was *H3* in all sample and 722B sample sets (Table 4). *ELF1A* was the most stably expressed gene with the lowest *SD* value in 722A sample set (Table 4).

**Table 4:** Stability ranking of 10 candidate reference gene using BestKeeper.

**Rank**	**All samples**		**722A**		**722B**	
	**Gene**	**SD**	**Gene**	**SD**	**Gene**	**SD**
1	*H3*	0.4	*ELF1A*	0.39	*H3*	0.21
2	*CYP*	0.66	*H3*	0.48	*GAPDH*	0.57
3	*TUB*	0.7	*CYP*	0.52	*18S*	0.64
4	*GAPDH*	0.96	*PEPKR1*	0.6	*CYP*	0.68
5	*PEPKR1*	0.97	*TUB*	0.7	*TUB*	0.69
6	*18S*	1.02	*18S*	0.93	*PEPKR1*	1.26
7	*ELF1A*	1.38	*ACT3*	1.22	*G6PD*	1.73
8	*G6PD*	1.9	*GAPDH*	1.33	*ELF1A*	2.28
9	*ACT3*	2.01	*G6PD*	2.08	*ACT3*	2.33
10	*TUA*	3.44	*TUA*	3.73	*TUA*	3.16

### Comprehensive stability ranking based on RefFinder

RefFinder integrates the above four statistical approaches to produce a comprehensive stability value for each candidate reference gene, and calculates the geometric mean of their ranking for the overall final ranking. For all sample set, *TUB, CYP* and *PEPKR* were the three most stable candidate reference genes, and the comprehensive stability rankings were: *TUB, CYP, PEPKR1, H3, 18S, ELF1A, GAPDH, ACT3, G6PD*, and *TUA* (Table 5; Table S2). For the 722A sample set, *ELF1A* and *H3* were the two most stable, and the comprehensive stability rankings were: *ELF1A, H3, TUB, PEPKR1, CYP, ACT3, 18S, GAPDH, G6PD*, and *TUA* (Table 5; Table S2). For the 722B sample set, *TUB* and *CYP* were the two most stable, and the comprehensive stability rankings were: *TUB, CYP, 18S, H3, GAPDH, PEPKR1, ELF1A, ACT3, G6PD*, and *TUA* (Table 5; Table S2).

**Table 5:** Comprehensive stability ranking of 10 candidate reference genes.

**Methods**	**1**	**2**	**3**	**4**	**5**	**6**	**7**	**8**	**9**	**10**
**ALL SAMPLES**										
RefFinder	*TUB*	*CYP*	*PEPKR1*	*H3*	*18S*	*ELF1A*	*GAPDH*	*ACT3*	*G6PD*	*TUA*
GeNorm	*TUB*/*CYP*	*PEPKR1*	*18S*	*H3*	*ELF1A*	*ACT3*	*GAPDH*	*G6PD*	*TUA*	
NormFinder	*TUB*	*CYP*	*PEPKR1*	*18S*	*ELF1A*	*H3*	*ACT3*	*G6PD*	*TUA*	*GAPDH*
ΔCt	*TUB*	*CYP*	*PEPKR1*	*H3*	*ELF1A*	*ACT3*	*GAPDH*	*G6PD*	*18S*	*TUA*
BestKeeper	*H3*	*CYP*	*TUB*	*GAPDH*	*PEPKR1*	*18S*	*ELF1A*	*G6PD*	*ACT3*	*TUA*
**722A**										
RefFinder	*ELF1A*	*H3*	*TUB*	*PEPKR1*	*CYP*	*ACT3*	*18S*	*GAPDH*	*G6PD*	*TUA*
GeNorm	*ELF1A*/*H3*	*TUB*	*PEPKR1*	*CYP*	*ACT3*	*18S*	*GAPDH*	*G6PD*	*TUA*	
NormFinder	*ELF1A*	*H3*	*TUB*	*PEPKR1*	*CYP*	*ACT3*	*18S*	*GAPDH*	*TUA*	*G6PD*
ΔCt	*H3*	*TUB*	*ELF1A*	*PEPKR1*	*CYP*	*18S*	*ACT3*	*GAPDH*	*G6PD*	*TUA*
BestKeeper	*ELF1A*	*H3*	*CYP*	*PEPKR1*	*TUB*	*18S*	*ACT3*	*GAPDH*	*G6PD*	*TUA*
**722B**										
RefFinder	*TUB*	*CYP*	*18S*	*H3*	*GAPDH*	*PEPKR1*	*ELF1A*	*ACT3*	*G6PD*	*TUA*
GeNorm	*TUB/CYP*	*18S*	*PEPKR1*	*H3*	*GAPDH*	*ELF1A*	*ACT3*	*G6PD*	*TUA*	
NormFinder	*TUB*	*CYP*	*18S*	*ELF1A*	*PEPKR*	*GAPDH*	*ACT3*	*H3*	*TUA*	*G6PD*
ΔCt	*TUB*	*CYP*	*18S*	*PEPKR1*	*GAPDH*	*H3*	*ELF1A*	*ACT3*	*G6PD*	*TUA*
BestKeeper	*H3*	*GAPDH*	*18S*	*CYP*	*TUB*	*PEPKR1*	*G6PD*	*ELF1A*	*ACT3*	*TUA*

### Reference gene validation

**Figure 5:**
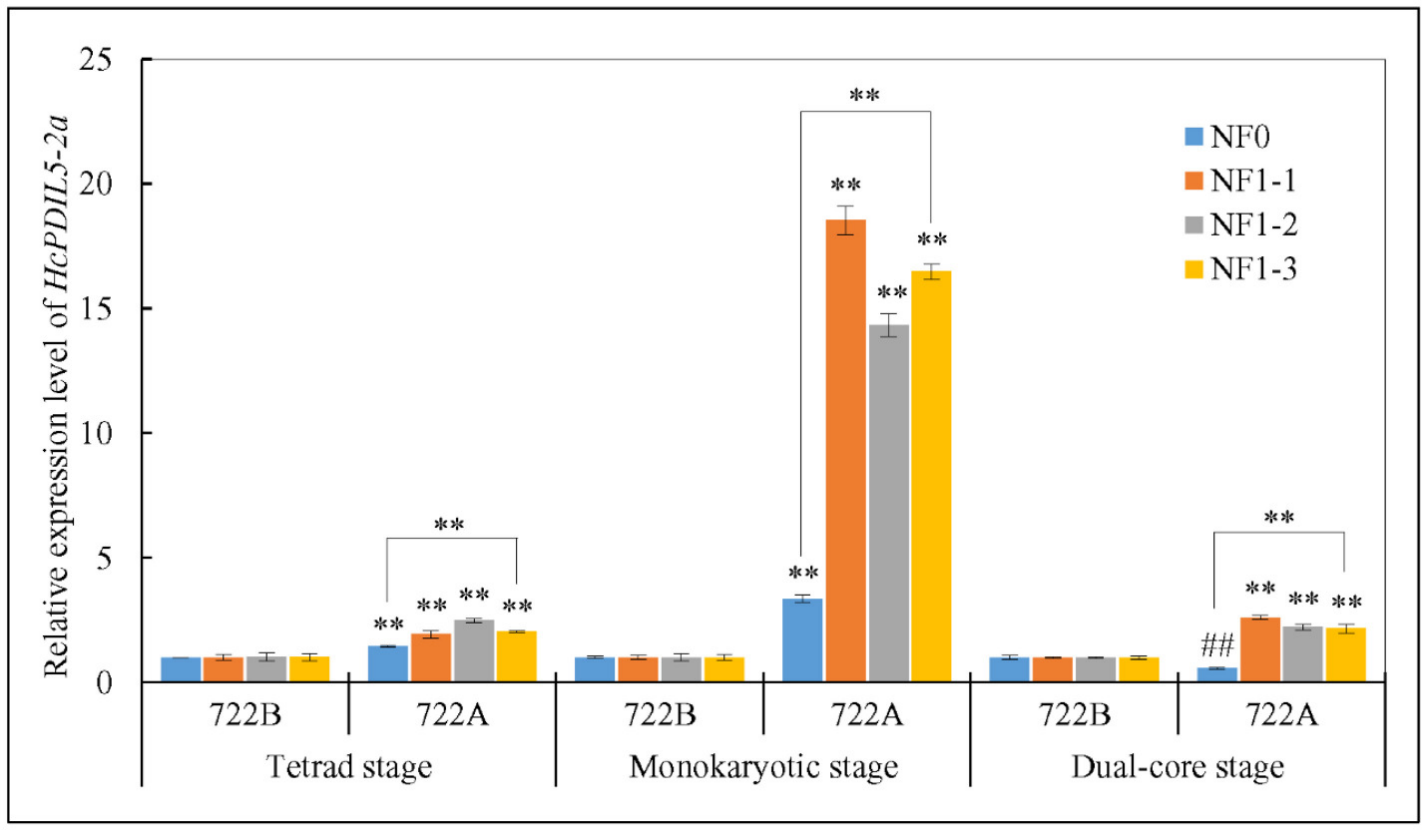
Relative expression levels of a target gene, *HcPDIL5-2a*, at tetrad, monokaryotic and dual-core stage in lines 722A and 722B using different normalization strategies. NF0: the least stable reference gene, *TUA*; NF1-1: the most stable reference gene, *TUB*; NF1-2: the most stable two reference gene, *TUB* and *CYP*; NF1-3: the most stable three reference gene, *TUB, CYP*, and *PEPKR1*; The error bars represent standard deviations of three biological replicates. Significantly up-regulated with respect to control was shown by ^**^P < 0.01, and significantly down-regulated was shown ^*##*^*P* < 0.01.

The relative expression level of the target gene *HcPDIL5-2a* was used to validate the reliability of the reference genes that were recommended by the RefFinder and GeNorm approaches. In the present study, the transcript levels of *HcPDIL5-2a* at three pollen developmental stages were normalized using four strategies: the one [NF1-1 (*TUB*)], two [NF1-2 (*TUB* and *CYP*)], and three [NF1-3 (*TUB, CYP* and *PEPKR1*)] most stable reference genes and the least stable reference gene [NF0 (*TUA*)] across all sample. At tetrad and monokaryotic stages, the expression levels of *HcPDIL5-2a* in line 722A were up-regulated relative to line 722B when normalized using NF1-1, NF1-2, NF1-3, and NF0. However, the expression levels of *HcPDIL5-2a* normalized using NF1-3 and NF0 were significantly different (*P* < 0.01) (Figure 5). When normalized using NF1-1, NF1-2, and NF1-3 at dual-core stage, the expression level of *HcPDIL5-2a* in line 722A was up-regulated compared with line 722B. In contrast, *HcPDIL5-2a*'s expression was down-regulated when normalized using NF0, and the expression levels of *HcPDIL5-2a* normalized using NF1-3 and NF0 were significantly different (*P* < 0.01) (Figure 5).

### Differential expression of mitochondria F_1_F_0_-ATPase subunit genes by RT-qPCR

**Figure 6:**
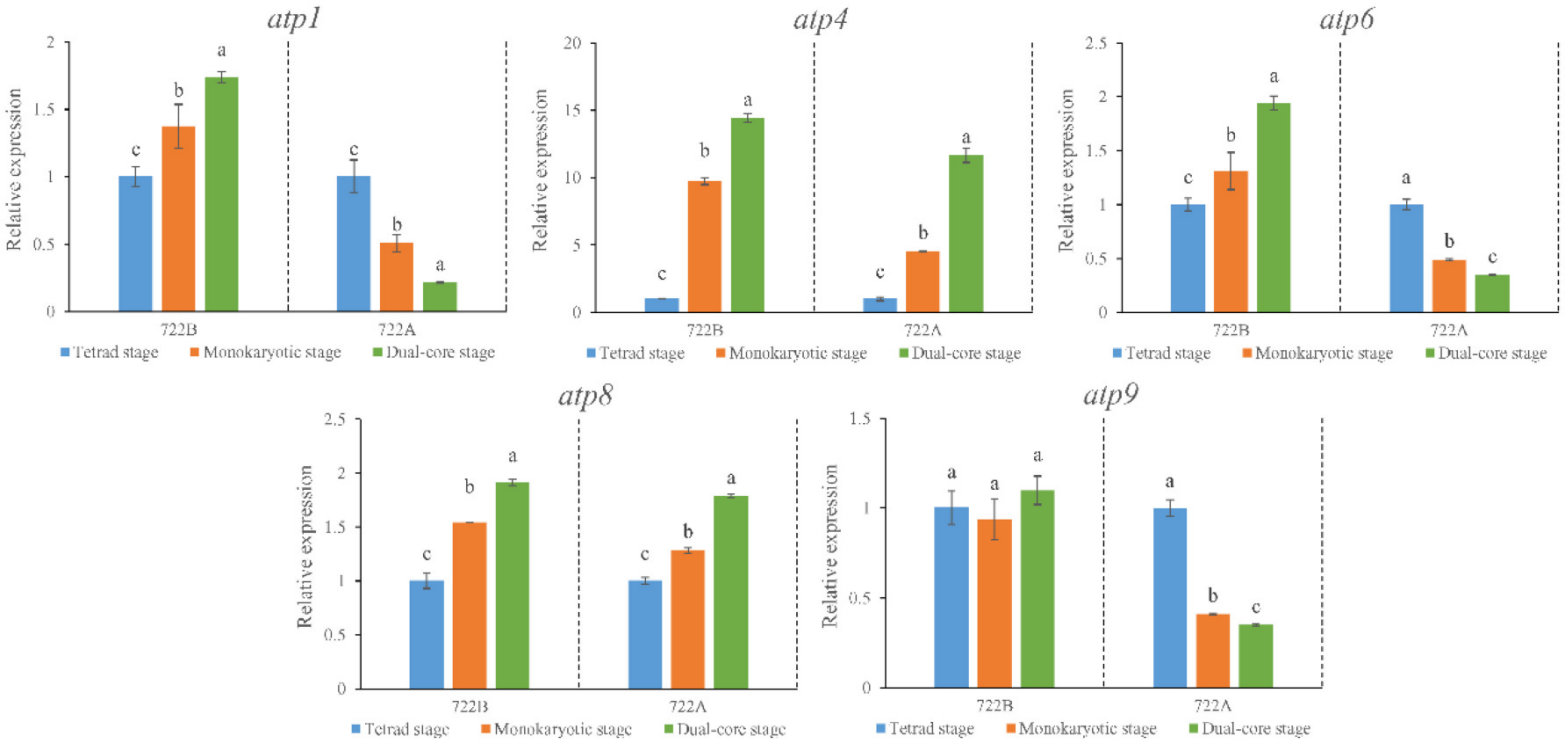
Expression pattern analysis of mitochondria F_1_F_0_-ATPase subunit genes *atp1, atp4, atp6, atp8, atp9*, using RT-qPCR at three stage of pollen development in lines 722A and 722B. The results were shown in relative expression level of the target genes normalized with the most stable three reference genes, *TUB, CYP*, and *PEPKR1*. Different lowercase letters indicate significant differences (*P* < 0.05) in relative expression of each gene among different pollen development stages. The error bars represent standard deviations of three biological replicates.

To explore the effects of the mitochondria F_1_F_0_-ATPase subunit genes (*atp1, atp4, atp6, atp8*, and *atp9*) on kenaf CMS, the expression patterns of these genes were differentiated during pollen development between lines 722A and 722B by RT-qPCR. All five genes (*atp1, atp4, atp6, atp8*, and *atp9*) exhibited a similar or significantly different expression pattern (Figure 6) in line 722A compared with line 722B after being normalized by the combination of *TUB, CYP*, and *PEPKR1*, which was recommend by GeNorm and RefFinder. The expression patterns of *atp1* and *atp6* were up-regulated gradually (*P* < 0.05) during pollen development in line 722B, but were down-regulated gradually (*P* < 0.05) in line 722A (Figure 6). During pollen development, *atp9* was down-regulated gradually (*P* < 0.05) in line 722A, whereas it maintained a steady level (*P* < 0.05) in line 722B (Figure 6). The expression patterns of *atp4* and *atp8* were increased (*P* < 0.05) in lines 722A and 722B (Figure 6).

## Discussion

The kenaf CMS line plays a critical role in F1 hybrid seed production and heterosis utilization. Analyses of the expression level of genes of interest that are involved in the regulation of pollen development would aid in our understanding of the molecular mechanisms of CMS in kenaf. RT-qPCR is a useful tool for gene expression studies that relies on a stable reference gene to normalize data. However, many studies have indicated that even the most stable reference gene cannot always be stably expressed under different environmental conditions (Artico et al., 2010). Therefore, it is necessary to assess and validate the stable reference genes under particular experimental conditions instead of using traditional reference genes that have been previously published. The present study is the first systematic survey of the expression stability of 10 candidate reference genes in a CMS and its maintainer lines of kenaf. The expression stability of these genes were evaluated using five statistical approaches, GeNorm, NormFinder, ΔCt, BestKeeper and RefFinder, to identify the most reliable reference genes.

The primer specificities of the target and reference genes should be checked first using several experimental approaches, such as agarose gel electrophoresis, sequencing and melting curves (Bustin et al., 2009; Wang S. et al., 2016). In this study, primer pairs for all 6 target genes and 10 candidate reference genes were validated by agarose gel electrophoresis (Figure S1), melting curve analysis (Figure S2) and sequencing. In addition, the primer *E* and *R*^2^ values were calculated using corresponding standard curves and ranged from 0.902 and 1.042, and 0.990 and 1.000, respectively. These results indicated that all of the material used were suitable for RT-qPCR analysis.

Previous research reported that the ranking order of stable reference genes through different statistic approaches (Wang C. et al., 2016). In this study, four commonly used statistic approaches (GeNorm, NormFinder, ΔCt, and BestKeeper) were used to evaluate the expression stabilities of candidate reference genes. The stability rankings of the 10 candidate reference genes were not the same when using different approaches (Table 5). For example, *TUB* was identified as the most stable reference gene by GeNorm, NormFinder and ΔCt in the sample sets, but *H3* was considered to be the most stable reference gene by BestKeeper. Similar results were observed in several studies (Zhang et al., 2015; Fan et al., 2016; Yue et al., 2016). Different statistical approaches, which are based on different principles, may yield contradictory results from the same data (Hu et al., 2009; Wan et al., 2010). This further confirms that a comprehensive approach (RefFinder) is necessary to evaluate the stability rankings of the 10 candidate reference genes.

It is has been well documented that the accuracy of RT-qPCR results can be significantly improved when multiple reference genes were used (Reid et al., 2006; Gutierrez et al., 2008; Cheng et al., 2017). Thus, in order to explore the optimal number of reference genes at different pollen development stages of kenaf, the Vn/n+1 values which were calculated by GeNorm approach. In this research, all of the Vn/n+1 values were above the proposed 0.15 cut-off across all of the sample (Figure 4D), which indicated that there was no optimal number of reference genes. However, many previous researchers have suggested that three of the most stable genes should be employed in situations when too many, or even no, optimal number of genes were determined (Kuijk et al., 2007; Silveira et al., 2009; Maroufi et al., 2010; Sun et al., 2015). Therefore, *TUB, CYP*, and *PEPKR1*, which were recommended by RefFinder, were used as internal controls.

Considering the comprehensive ranking, *TUB* was identified overall as the most stable candidate reference gene across all of the sample sets. Tubulin plays a critical role in structural support, intracellular transport and DNA segregation. In previously studies, *TUB* has been confirmed as the most reliable reference genes in CMS7311 and female cabbage during flower development and in *Platycladus orientalis* under stress conditions (Chang et al., 2012; Xu et al., 2014). By contrast, *TUB* has been excluded as a good internal control in the sexual tissues of *Brachiaria brizantha* (Silveira et al., 2009). *TUA* was the least stable candidate reference gene based on the RefFinder approach in this study, indicating that this gene should be avoided as an internal control when analyzing gene expression patterns in CMS and maintainer lines during pollen development. Our validation experiment indicated that the use of *TUA* as an internal control led to the misinterpretation of the *HcPDIL5-2a* expression levels at different stages of pollen development in lines 722A and 722B (Figure 5). By contrast, *TUA* exhibited a high stability during seed development in peanut (Chi et al., 2012).

*GAPDH* encodes an important catalyzing enzyme that is involved in the sixth step of glycolysis and has several functions in non-metabolic processes (Tarze et al., 2007; Zala et al., 2013). It was considered as the most stable reference gene under various experimental conditions (Qi et al., 2010) and has been extensively used as a reference gene in kenaf (Zhao et al., 2013; Liao et al., 2016). In contrast, our results indicated that *GAPDH* was only moderately stable and was not an ideal reference gene for gene expression analyses during pollen development in lines 722A and 722B (Table 3). It is well-known that pollen development/abortion is related to energy metabolism and energy deficiency. The above results may be caused by *GAPDH*'s involvement in energy metabolism, eventually leading to a decrease in expression stability.

Previous studies showed that the application of invalidated reference genes may significantly affect the quantifications of gene expression analyses (Lovdal and Saha, 2014). Even using the single most stable reference gene after assessment, may result in contradictory outcomes (Ding et al., 2015). In the present study, the levels of *HcPDIL5-2a* expression varied substantially after normalizing with one, two and three of the most and least stable reference genes (Figure 5). The results indicated that using unstable reference genes or a single reference gene is insufficient to obtain reliable and accurate results. Therefore, the expression stability of reference genes should be assessed and multiple reference genes should be used under particular experimental conditions to obtain more accurate and reliable results (Zhu et al., 2012).

Additionally, the combination of *TUB, CYP*, and *PEPKR1* as an internal control was used to normalize the expression pattern of five mitochondria F_1_F_0_-ATPase subunit genes (*atp1, atp4, atp6, atp8*, and *atp9*) in lines 722A and 722B. These genes are involved in energy metabolism in plant cells. Their expression patterns in line 722A provided a preliminary impression of the complicated CMS molecular mechanisms in kenaf.

Pollen development is highly energy-consuming process (Lee and Warmke, 1979). Mitochondria are the site of both the tricarboxylic acid (TCA) and oxidative phosphorylation pathway, plays a crucial role in energy and carbon metabolism in eukaryotic cells (Hatefi, 1985). A vital component of all mitochondria is F_1_F_0_-ATPase (Complex V) which is reversibly involved in the synthesis and the hydrolysis of adenosine triphosphate (ATP), depending upon the direction of an electrochemical gradient that is formed by the passage of protons through F_1_F_0_-ATPase (Senior, 1979; Yesodi et al., 1997). The abnormal expression of F_1_F_0_-ATPase subunit gene could cause destruction of membrane electronic potential, which led to mitochondria dysfunction and eventually pollen defect (Li et al., 2010). In the present study, five subunit genes (*atp1, atp4, atp6, atp8*, and *atp9*) of mitochondria F_1_F_0_-ATPase had significantly increased or stable expression pattern in line 722B (Figure 6), indicating that these genes had basic function under pollen development for maintaining the normal membrane electronic potential. In contrast, three subunit genes (*atp1, atp6*, and *atp9*) of mitochondria F_1_F_0_-ATPase exhibited gradually down-regulated expression patterns in line 722A, indicating that the normal membrane electronic potential may be was destroyed, which led to mitochondria dysfunction and eventually pollen defect in kenaf. However, it is necessary to study them further and to verify their correlation with kenaf' CMS.

## Conclusion

This study evaluated the expression stability of 10 candidate reference genes across 19 samples using five common statistical approaches. A gene combination (*TUB, CYP*, and *PEPKR1*) was predicted to be reliable and was validated using the reference genes to normalize the expression levels of *HcPDIL5-2a* using RT-qPCR, during the pollen development stage in lines 722A and 722B. Furthermore, the combination of *TUB, CYP*, and *PEPKR1* was used as an internal control for differentiating the expression patterns of five mitochondria F1F0-ATPase subunit genes (*atp1, atp4, atp6, atp8*, and *atp9*) during the pollen development in lines 722A and 722B. Three genes (*atp1, atp6*, and *atp9*) exhibited significant expression trends in the line 722A compared with line 722B. The data suggest that these genes may be associated with the CMS of kenaf crop. These data will provide useful information for future research on gene expression analysis.

## Author contributions

RZ conceived and designed the research. PC and AK revised the manuscript. BZ, YZ, and DL analyzed data and wrote the manuscript; LC, XL, and XK assist during the experiments. All authors read and approved the manuscript.

### Conflict of interest statement

The authors declare that the research was conducted in the absence of any commercial or financial relationships that could be construed as a potential conflict of interest.
