# Supplementary material for: Candidate Reference Genes Selection and Application for RT-qPCR Analysis in Kenaf with Cytoplasmic Male Sterility Background
Source: Front Plant Sci. 2017 Sep 1;8:1520. doi: 10.3389/fpls.2017.01520 (PMC5585197; doi:10.3389/fpls.2017.01520)
Supplement: Supplementary file 1 [file Table1.DOCX]

**Table S1.** The Ct values of 10 candidate reference genes

| 722B dual-core stage |  |  | ***18S*** | ***ACT3*** | ***CYP*** | ***EFL1A*** | ***G6PD*** | ***GAPDH*** | ***H3*** | ***PEPKR1*** | ***TUA*** | ***TUB*** |
| --- | --- | --- | --- | --- | --- | --- | --- | --- | --- | --- | --- | --- |
|  | Sample replicate1 | Technical replicate 1 | 7.17 | 19.84 | 20.13 | 20.9 | 27.13 | 24.53 | 21.5 | 25.66 | 24.45 | 27.45 |
|  |  | Technical replicate 2 | 7.15 | 19.82 | 20.25 | 21.04 | 27.35 | 24.47 | 21.39 | 26.12 | 24.72 | 27.29 |
|  |  | Technical replicate 3 | 7.13 | 19.2 | 19.86 | 20.79 | 26.7 | 24.31 | 21.44 | 25.27 | 24.47 | 27.27 |
|  | Sample replicate2 | Technical replicate 1 | 7.26 | 19.18 | 20.14 | 21.1 | 27.28 | 24.6 | 21.48 | 25.39 | 25.08 | 27.41 |
|  |  | Technical replicate 2 | 7.12 | 18.81 | 20.5 | 21.3 | 27.11 | 24.7 | 21.38 | 24.82 | 25.05 | 27.38 |
|  |  | Technical replicate 3 | 7.14 | 19.54 | 20.16 | 21.02 | 26.98 | 24.83 | 21.63 | 25.92 | 24.93 | 27.49 |
|  | Sample replicate3 | Technical replicate 1 | 7.25 | 19.32 | 19.97 | 20.85 | 26.59 | 24.58 | 21.66 | 25.24 | 24.56 | 27.55 |
|  |  | Technical replicate 2 | 7.15 | 19 | 19.95 | 21.01 | 26.92 | 24.77 | 21.4 | 24.68 | 24.5 | 27.15 |
|  |  | Technical replicate 3 | 7.1 | 19.36 | 19.82 | 20.79 | 26.9 | 24.79 | 21.48 | 25.42 | 24.64 | 27.31 |
| 722A dual-core stage | Sample replicate1 | Technical replicate 1 | 8.68 | 21.54 | 19.94 | 23.64 | 27.11 | 25.06 | 20.38 | 25.39 | 22.45 | 27.75 |
|  |  | Technical replicate 2 | 8.55 | 21.57 | 19.75 | 23.4 | 26.96 | 25 | 20.44 | 25.23 | 22.85 | 27.46 |
|  |  | Technical replicate 3 | 8.8 | 21.57 | 19.94 | 23.34 | 27.26 | 25.07 | 20.54 | 25.34 | 22.93 | 27.61 |
|  | Sample replicate2 | Technical replicate 1 | 8.84 | 22.02 | 20.08 | 23.78 | 27.45 | 25.02 | 20.58 | 25.49 | 23.07 | 27.89 |
|  |  | Technical replicate 2 | 8.96 | 22.12 | 20.22 | 23.97 | 27.52 | 25.21 | 20.34 | 25.87 | 22.7 | 27.72 |
|  |  | Technical replicate 3 | 8.88 | 22.01 | 20.14 | 23.91 | 27.66 | 25.34 | 20.36 | 25.43 | 23 | 27.73 |
|  | Sample replicate3 | Technical replicate 1 | 8.71 | 21.14 | 20.05 | 23.48 | 27.48 | 25.32 | 20.76 | 25.18 | 22.49 | 27.49 |
|  |  | Technical replicate 2 | 8.77 | 21.32 | 19.96 | 23.35 | 27.2 | 25.31 | 20.65 | 25.05 | 22.69 | 28.04 |
|  |  | Technical replicate 3 | 8.84 | 21.55 | 19.95 | 23.41 | 27.28 | 25.39 | 20.69 | 25.09 | 22.46 | 27.48 |
| 722B monokaryotic stage | Sample replicate1 | Technical replicate 1 | 6.44 | 20.53 | 19.32 | 22.06 | 22.64 | 25.91 | 21.49 | 24.45 | 29.99 | 27.06 |
|  |  | Technical replicate 2 | 6.52 | 21.25 | 19.37 | 22.49 | 23.07 | 26.01 | 21.56 | 24.57 | 30.26 | 27.08 |
|  |  | Technical replicate 3 | 6.67 | 21.04 | 19.54 | 22.17 | 22.52 | 26.12 | 21.38 | 24.28 | 30.61 | 26.82 |
|  | Sample replicate2 | Technical replicate 1 | 6.4 | 20.85 | 19.79 | 22.24 | 22.9 | 25.97 | 21.5 | 24.5 | 29.89 | 27.21 |
|  |  | Technical replicate 2 | 6.42 | 20.99 | 19.81 | 22.3 | 22.73 | 25.94 | 21.88 | 24.61 | 29.84 | 27.09 |
|  |  | Technical replicate 3 | 6.51 | 21.1 | 19.54 | 22.28 | 22.72 | 26.15 | 21.63 | 24.57 | 30.42 | 26.98 |
|  | Sample replicate3 | Technical replicate 1 | 6.44 | 21.29 | 19.95 | 22.46 | 23.07 | 26.28 | 21.86 | 24.76 | 30.3 | 27.22 |
|  |  | Technical replicate 2 | 6.06 | 21.28 | 19.92 | 22.68 | 23.18 | 26.18 | 21.85 | 24.86 | 30.39 | 27.3 |
|  |  | Technical replicate 3 | 6.15 | 21.17 | 19.99 | 22.58 | 22.79 | 26.37 | 21.81 | 24.5 | 29.93 | 27.13 |
| 722A monokaryotic stage | Sample replicate1 | Technical replicate 1 | 10.41 | 24.88 | 21.07 | 24.07 | 23.98 | 24.52 | 21.55 | 27.18 | 29.9 | 29.39 |
|  |  | Technical replicate 2 | 10.09 | 25.27 | 21.16 | 24.12 | 24 | 24.48 | 21.48 | 27 | 29.94 | 29.52 |
|  |  | Technical replicate 3 | 10.19 | 24.61 | 21.38 | 24.31 | 24.04 | 24.83 | 21.54 | 27.36 | 30.14 | 29.79 |
|  | Sample replicate2 | Technical replicate 1 | 9.97 | 24.9 | 21.26 | 24.44 | 24.27 | 24.96 | 22.16 | 26.9 | 30.21 | 28.96 |
|  |  | Technical replicate 2 | 10.22 | 25 | 21.31 | 24.67 | 24.48 | 25.28 | 21.59 | 27.14 | 30.28 | 29.17 |
|  |  | Technical replicate 3 | 10.29 | 25.49 | 21.34 | 24.78 | 24.31 | 25.61 | 21.84 | 27.04 | 30.3 | 29.15 |
|  | Sample replicate3 | Technical replicate 1 | 9.41 | 23.61 | 21.12 | 24.35 | 23.72 | 25.03 | 21.6 | 26.91 | 29.76 | 29.35 |
|  |  | Technical replicate 2 | 9.39 | 25.58 | 21.48 | 24.68 | 24.41 | 25.14 | 21.65 | 26.93 | 29.5 | 29.44 |
|  |  | Technical replicate 3 | 9.73 | 25.82 | 21.16 | 24.1 | 24.21 | 25.39 | 22.1 | 27.11 | 30.48 | 29.19 |
| 722B tetrad stage | Sample replicate1 | Technical replicate 1 | 8.11 | 25.28 | 21.72 | 26.68 | 23.35 | 25.69 | 21.16 | 27.92 | 33.17 | 29.05 |
|  |  | Technical replicate 2 | 8.15 | 25.43 | 21.72 | 26.84 | 23.42 | 25.34 | 21.11 | 28.02 | 33.38 | 29 |
|  |  | Technical replicate 3 | 8.06 | 25.2 | 21.59 | 26.81 | 23.15 | 25.27 | 21.1 | 27.92 | 33.2 | 28.88 |
|  | Sample replicate2 | Technical replicate 1 | 8.25 | 25.39 | 21.76 | 27.13 | 23.36 | 26.04 | 21.2 | 28.09 | 33.29 | 29.13 |
|  |  | Technical replicate 2 | 8.52 | 26.04 | 21.35 | 26.76 | 23.47 | 25.62 | 21.05 | 27.91 | 33.79 | 28.76 |
|  |  | Technical replicate 3 | 8.34 | 26.11 | 21.17 | 26.95 | 23.89 | 25.72 | 20.95 | 27.41 | 33.74 | 28.44 |
|  | Sample replicate3 | Technical replicate 1 | 8.01 | 25.03 | 21.02 | 26.62 | 23.21 | 25.84 | 21.22 | 27.55 | 33.52 | 28.43 |
|  |  | Technical replicate 2 | 8.31 | 25.01 | 21.08 | 26.69 | 23.15 | 25.49 | 21.15 | 27.55 | 33.6 | 28.59 |
|  |  | Technical replicate 3 | 8.19 | 25.49 | 21.17 | 26.8 | 23.25 | 25.55 | 21.21 | 27.64 | 33.44 | 28.72 |
| 722A tetrad stage | Sample replicate1 | Technical replicate 1 | 7.22 | 23.21 | 20.98 | 23.34 | 21.1 | 22.03 | 20.41 | 26.28 | 31.9 | 27.7 |
|  |  | Technical replicate 2 | 7.29 | 23.45 | 21.16 | 23.55 | 21.24 | 21.82 | 20.58 | 26.23 | 32.43 | 27.62 |
|  |  | Technical replicate 3 | 7.19 | 23.35 | 21.05 | 23.43 | 21.22 | 22.24 | 20.94 | 26.19 | 32.51 | 27.48 |
|  | Sample replicate2 | Technical replicate 1 | 7.01 | 23.88 | 21.11 | 23.32 | 21.28 | 22.28 | 20.8 | 26.19 | 32.19 | 27.83 |
|  |  | Technical replicate 2 | 7.1 | 23.77 | 21.07 | 23.44 | 21.12 | 22.04 | 20.84 | 26.12 | 31.82 | 27.98 |
|  |  | Technical replicate 3 | 7.18 | 23.67 | 21.1 | 24.02 | 21.24 | 22.07 | 21.1 | 26.32 | 32.41 | 27.85 |
|  | Sample replicate3 | Technical replicate 1 | 7.51 | 23.9 | 21.11 | 23.73 | 21.48 | 22.25 | 21.05 | 26.39 | 32.49 | 27.94 |
|  |  | Technical replicate 2 | 7.54 | 23.64 | 21.17 | 23.72 | 21.37 | 22.15 | 20.8 | 26.36 | 31.68 | 28.05 |
|  |  | Technical replicate 3 | 7.61 | 24.16 | 21.11 | 23.62 | 21.48 | 22.22 | 20.62 | 26.24 | 32.34 | 27.88 |
