# Supplementary material for: Candidate Reference Genes Selection and Application for RT-qPCR Analysis in Kenaf with Cytoplasmic Male Sterility Background
Source: Front Plant Sci. 2017 Sep 1;8:1520. doi: 10.3389/fpls.2017.01520 (PMC5585197; doi:10.3389/fpls.2017.01520)
Supplement: Supplementary file 2 [file Table2.DOCX]

| Samples | Comprehensive ranking（ReFinder） | |  | GeNorm | |  | NormFinder | |  | △Ct | |  | BestKeeper | |
| --- | --- | --- | --- | --- | --- | --- | --- | --- | --- | --- | --- | --- | --- | --- |
|  |  |  |  |  |  |  |  |  |  |  |  |  |  |  |
|  | Gene | Geometric mean |  | Gene | M-value |  | Gene | Stability value |  | Gene | Mean SD |  | Gene | SD |
|  |  |  |  |  |  |  |  |  |  |  |  |  |  |  |
| All samples | *TUB* | 1.32 |  | *TUB* | *0.44* |  | *TUB* | 0.11 |  | *TUB* | 1.49 |  | *H3* | 0.4 |
|  | *CYP* | 1.68 |  | *CYP* | 0.44 |  | *CYP* | 0.11 |  | *CYP* | 1.54 |  | *CYP* | 0.66 |
|  | *PEPKR1* | 3.08 |  | *PEPKR1* | 0.51 |  | *PEPKR1* | 0.14 |  | *PEPKR1* | 1.55 |  | *TUB* | 0.69 |
|  | *H3* | 3.13 |  | *18S* | 0.73 |  | *18S* | 0.18 |  | *H3* | 1.80 |  | *GAPDH* | 0.96 |
|  | *18S* | 5.05 |  | *H3* | 0.87 |  | *ELF1A* | 0.19 |  | *ELF1A* | 1.92 |  | *PEPKR1* | 0.97 |
|  | *ELF1A* | 5.44 |  | *ELF1A* | 1.08 |  | *H3* | 0.20 |  | *ACT3* | 2.16 |  | *18S* | 1.02 |
|  | *GAPDH* | 6.65 |  | *ACT3* | 1.24 |  | *ACT3* | 0.20 |  | *GAPDH* | 2.16 |  | *ELF1A* | 1.36 |
|  | *ACT3* | 6.90 |  | *GAPDH* | 1.39 |  | G6PD | 0.22 |  | G6PD | 3.13 |  | G6PD | 1.89 |
|  | *G6PD* | 8.00 |  | G6PD | 1.7 |  | *TUA* | 0.22 |  | *18S* | 3.50 |  | *ACT3* | 2.01 |
|  | *TUA* | 9.49 |  | *TUA* | 2.15 |  | *GAPDH* | 0.25 |  | *TUA* | 3.95 |  | *TUA* | 3.44 |
| 722A | *H3* | 1.19 |  | *ELF1A* | 0.26 |  | *H3* | 0.05 |  | *H3* | 1.30 |  | *ELF1A* | 0.37 |
|  | *ELF1A* | 1.73 |  | *H3* | 0.26 |  | *TUB* | 0.06 |  | *TUB* | 1.32 |  | *H3* | 0.47 |
|  | *TUB* | 2.51 |  | *TUB* | 0.36 |  | *ELF1A* | 0.07 |  | *ELF1A* | 1.33 |  | *CYP* | 0.52 |
|  | *PEPKR1* | 3.72 |  | *PEPKR1* | 0.39 |  | *PEPKR1* | 0.17 |  | *PEPKR1* | 1.34 |  | *PEPKR1* | 0.59 |
|  | *CYP* | 4.16 |  | *CYP* | 0.42 |  | *CYP* | 0.22 |  | *CYP* | 1.42 |  | *TUB* | 0.70 |
|  | *ACT3* | 6.19 |  | *ACT3* | 0.60 |  | *ACT3* | 0.28 |  | *18S* | 1.60 |  | *18S* | 0.93 |
|  | *18S* | 6.24 |  | *18S* | 0.74 |  | *18S* | 0.28 |  | *ACT3* | 1.74 |  | *ACT3* | 1.22 |
|  | *GAPDH* | 7.97 |  | *GAPDH* | 0.95 |  | *TUA* | 0.40 |  | *GAPDH* | 1.99 |  | *GAPDH* | 1.33 |
|  | *G6PD* | 8.97 |  | G6PD | 1.35 |  | *GAPDH* | 0.41 |  | *G6PD* | 3.20 |  | *G6PD* | 2.07 |
|  | *TUA* | 9.21 |  | *TUA* | 1.97 |  | G6PD | 0.46 |  | *TUA* | 4.46 |  | *TUA* | 3.73 |
| 722B | *TUB* | 1.50 |  | *TUB* | 0.13 |  | *TUB* | 0.12 |  | *TUB* | 1.42 |  | *H3* | 0.20 |
|  | *CYP* | 2.00 |  | *CYP* | 0.13 |  | *CYP* | 0.13 |  | *CYP* | 1.44 |  | *GAPDH* | 0.55 |
|  | *18S* | 2.71 |  | *18S* | 0.25 |  | *18S* | 0.21 |  | *18S* | 1.50 |  | *18S* | 0.64 |
|  | *H3* | 3.72 |  | *PEPKR1* | 0.48 |  | *GAPDH* | 0.25 |  | *PEPKR1* | 1.60 |  | *CYP* | 0.67 |
|  | *GAPDH* | 3.76 |  | *H3* | 0.76 |  | *PEPKR1* | 0.26 |  | *GAPDH* | 1.83 |  | *TUB* | 0.69 |
|  | *PEPKR1* | 4.36 |  | *GAPDH* | 0.88 |  | *ELF1A* | 0.27 |  | *H3* | 1.91 |  | *PEPKR1* | 1.24 |
|  | *ELF1A* | 6.70 |  | *ELF1A* | 1.24 |  | *ACT3* | 0.29 |  | *ELF1A* | 2.11 |  | *G6PD* | 1.73 |
|  | *ACT3* | 7.71 |  | *ACT3* | 1.42 |  | *H3* | 0.32 |  | *ACT3* | 2.18 |  | *ELF1A* | 2.28 |
|  | *G6PD* | 8.43 |  | *G6PD* | 1.72 |  | *TUA* | 0.39 |  | *G6PD* | 3.09 |  | *ACT3* | 2.33 |
|  | *TUA* | 9.49 |  | *TUA* | 2.04 |  | *G6PD* | 0.54 |  | *TUA* | 3.33 |  | *TUA* | 3.16 |

**Table S2**. Gene expression stability ranked by comprehensive ranking, GeNorm, NormFinder, △Ct and BestKeeper in kenaf.
