# Supplementary material for: Candidate Reference Genes Selection and Application for RT-qPCR Analysis in Kenaf with Cytoplasmic Male Sterility Background
Source: Front Plant Sci. 2017 Sep 1;8:1520. doi: 10.3389/fpls.2017.01520 (PMC5585197; doi:10.3389/fpls.2017.01520)
Supplement: Supplementary file 3 [file Image1.PDF]

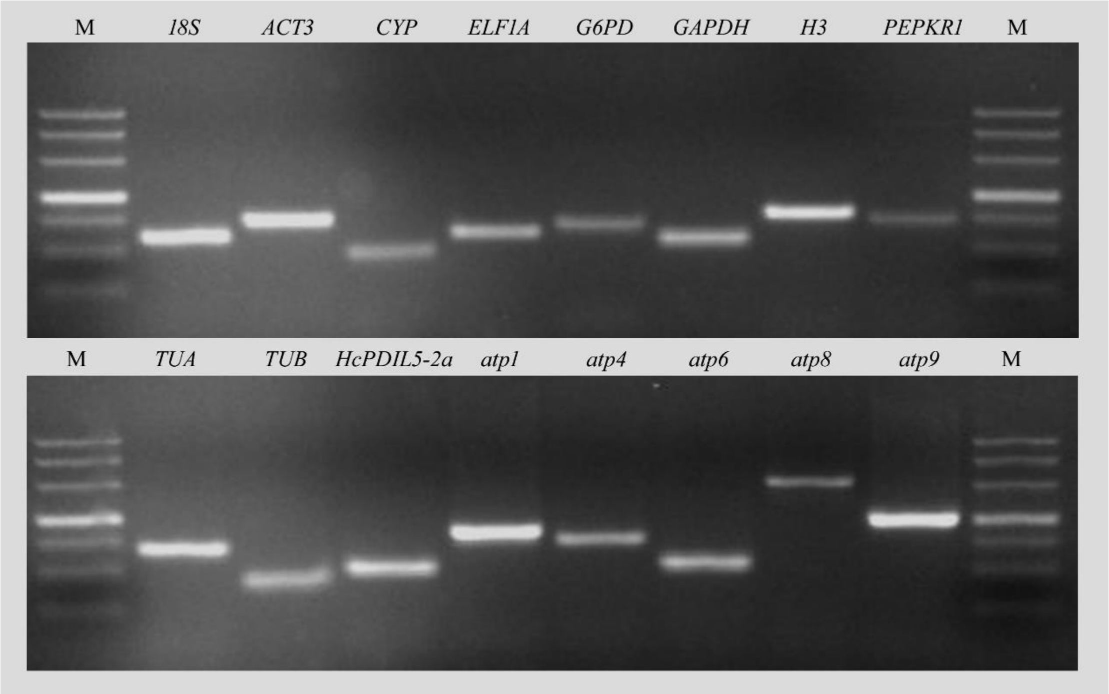

Fig. S1 Amplified products of 10 candidate reference genes and 6 target genes were separated by 3% Agarose gel electrophoresis. M: DL500 marker.
