# Supplementary material for: Candidate Reference Genes Selection and Application for RT-qPCR Analysis in Kenaf with Cytoplasmic Male Sterility Background
Source: Front Plant Sci. 2017 Sep 1;8:1520. doi: 10.3389/fpls.2017.01520 (PMC5585197; doi:10.3389/fpls.2017.01520)
Supplement: Supplementary file 4 [file Image2.PDF]

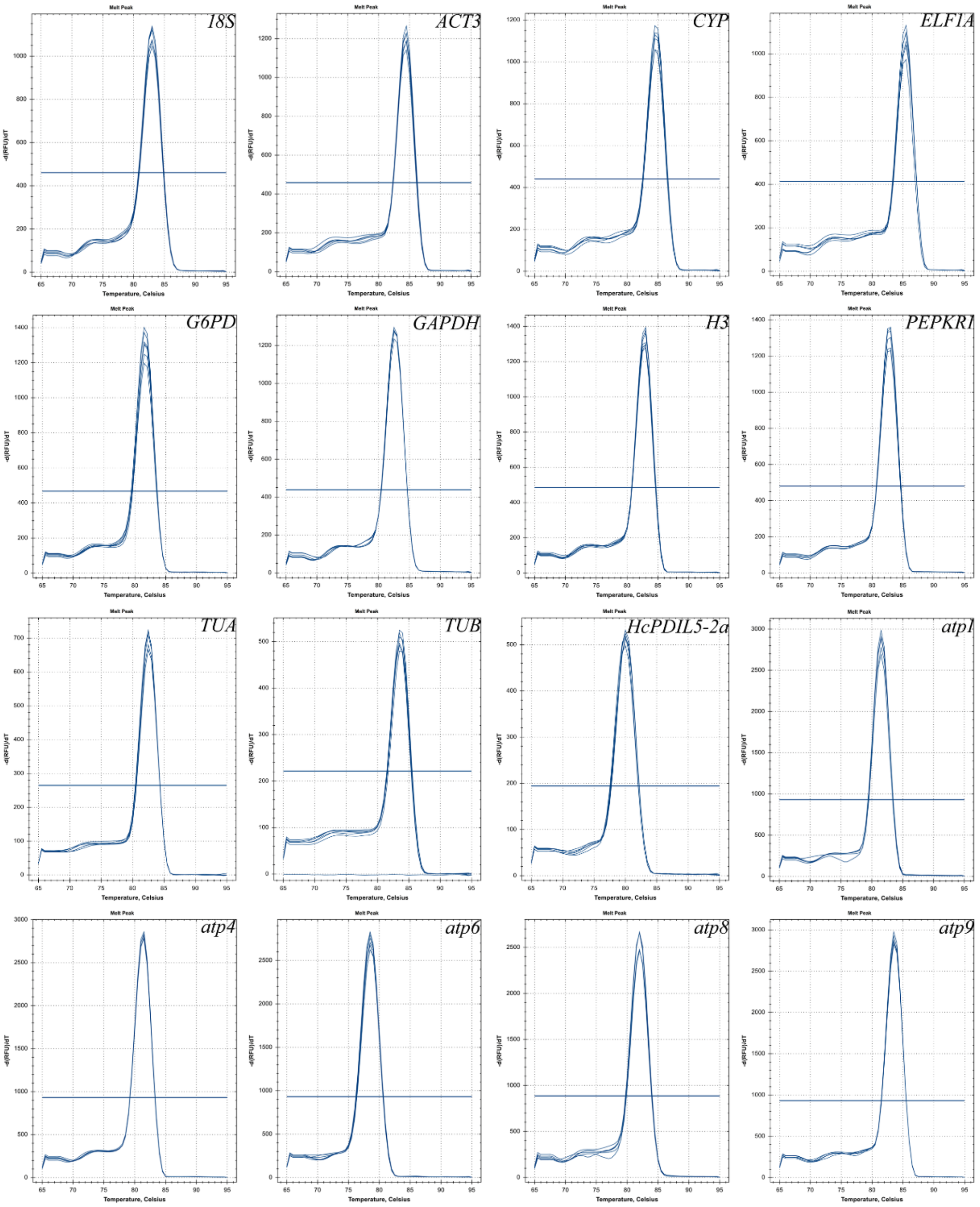

Fig. S2 Melting curves of 10 candidate reference genes and six target genes in kenaf. Melting temperatures were visualized by plotting the negative first derivative of fluorescence relative to the temperature in Celsius [-(d/dT)].
